# Effectiveness of HCV core antigen and RNA quantification in HCV-infected and HCV/HIV-1-coinfected patients

**DOI:** 10.1186/s12879-014-0577-1

**Published:** 2014-11-05

**Authors:** Lu Long, Tao Shen, Jian Gao, Zhaojun Duan, Hua Liang, Fengmin Lu

**Affiliations:** Department of Microbiology and Center of Infectious Diseases, Peking University Health Science Center, 38 Xueyuan Road, Haidian District, Beijing, 100191 China; State Key Laboratory for Infectious Disease Prevention and Control, National Center for AIDS/STD Control and Prevention, China CDC, Collaborative Innovation Center for Diagnosis and Treatment of Infectious Diseases, Beijing, 100026 China

**Keywords:** CD4+ T-cell counts, Coinfection, HCV-coreAg, HCV-RNA, HCV/HIV-1, Ratio

## Abstract

**Background:**

The measurement of hepatitis C virus core antigen (HCV-coreAg) has been shown to be an indicator of active HCV infection. The aim of the present study was 1) to investigate the stability and effectiveness of HCV-coreAg and HCV-RNA quantification in HCV infection with or without HIV-1 coinfection, 2) to explore the association between the HCV-coreAg/HCV-RNA (Ag/RNA) ratio and the immune status in chronic HCV/HIV-1-coinfected patients.

**Methods:**

A longitudinal investigation comprised of 227 HCV-monoinfected (n = 129) and HCV/HIV-1-coinfected (n = 98) patients was initiated in August 2009, and 139 (73 with HCV monoinfection and 66 with HCV/HIV-1 coinfection) were followed up in August 2012. Both HCV core antigen and HCV RNA quantification were determined on this cryopreserved plasma. HCV core antigen and HCV RNA quantification were performed subsequently. In addition, an *in vitro* experiment investigating the possibility of degradation of HCV components (core antigen and RNA) were conducted.

**Results:**

Significant and stable correlations (p < 0.001) were observed both in chronic HCV-monoinfected and HCV/HIV-1-coinfected patients over the 3-year observation. Coinfected patients with immunocompromised condition had a significantly higher (p < 0.05) Ag/RNA ratios than those patients with immunocompetent condition both at two time points (2009 and 2012). Moreover, the Ag/RNA ratios were negatively correlated with CD4+ T-cell counts (p < 0.001). An *in vitro* experiment investigated the possibility of the slower degradation of HCV particles under HIV-related immunocompromised condition was conducted and the data demonstrated that the Ag/RNA ratios were significantly higher in HIV-1-positive plasma than in healthy plasma (p = 0.005) in this study.

**Conclusions:**

Our longitudinal study indicated that the HCV-coreAg presented comparable dynamics over time as HCV RNA in chronic HCV-infected patients. Meanwhile, the HCV-coreAg/HCV-RNA ratio was closely associated with immune status in HCV/HIV-1-coinfected patients.

**Electronic supplementary material:**

The online version of this article (doi:10.1186/s12879-014-0577-1) contains supplementary material, which is available to authorized users.

## Background

Hepatitis C virus (HCV) is one of the major causes of liver cirrhosis and hepatocellular carcinoma [1]. Approximately 170 million individuals around the world are chronically infected with HCV and 34 million individuals currently suffer from human immunodeficiency virus (HIV) [2]-[4]. HCV/HIV-1 coinfection is common due to their shared routes of transmission. In China, a higher prevalence rate of coinfection in several provinces has been linked to unsanitary commercial blood [4]-[7]. In clinical practice, as a routine screening test for the diagnosis of HCV infection, anti-HCV assay has the disadvantage of failing to distinguish between active infection and prior infection that has resolved spontaneously or after antiviral treatment. In addition, false positive anti-HCV tests are often found in pregnant women and patients with nephrological or rheumatoid diseases [8]. On the other hand, our previous observation [9] hints false negative anti-HCV results are becoming more frequent in immunocompromised patients, particularly those with AIDS.

Real-time RT-PCR is an alternative strategy that does not have this disadvantage. However, detection of HCV-RNA by real-time RT-PCR requires skill, is time-consuming, and is too expensive for primary hospitals, despite its merit in evaluating HCV replication [10],[11].

Another cheaper alternative in lieu of HCV-RNA detection is quantification of hepatitis C virus core antigen (HCV-coreAg). Our previous study [9] and others [12]-[17] have widely investigated the potential applicability of this strategy to the diagnosis of active HCV infection and monitoring of antiviral response, due to its correlation with RT-PCR, as well as its ease of automation. Despite these advantages, the further improvement for the detection limit of HCV-coreAg assay is still highly desired [8]. Recently, Garbuglia et al. [18] demonstrated that HCV-coreAg also represent an adequate tool for determining an active HCV infection in HCV/HIV coinfected patients with different HCV genotyping. The present report describes a 3-year longitudinal study to further investigate the stability and effectiveness of HCV-coreAg and HCV-RNA quantification in HCV infection with or without HIV-1 coinfection.

## Methods

### Study population

**Figure 1:**
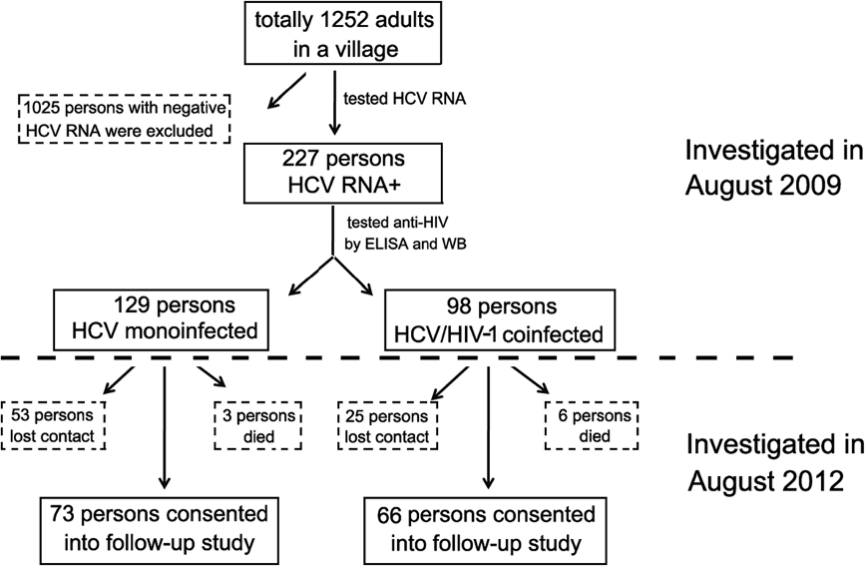
**A flow diagram for recruited persons in the study.**

The study, a longitudinal investigation of 227 HCV-monoinfected (129 persons) and HCV/HIV-1-coinfected (98 persons) patients, was initiated in August 2009. The patients were residents of a village in Shangcai county, Henan province in central China, with an adult population of 1,252. A high prevalence rate of blood-borne AIDS and chronic hepatitis C in the same village was reported previously [19]. Of the 227 patients originally enrolled, 139 (73 with HCV monoinfection and 66 with HCV/HIV-1 coinfection) were followed up in August 2012; the other 88 were lost because of death or loss of contact. All the 139 persons were confirmed with positive reaction of plasma HCV RNA tests and no subject was found to turn HCV RNA negative at the end of follow-up. A flow diagram for recruited persons is illustrated in Figure 1. HCV infection was defined by positive anti-HCV response and positive results for HCV-RNA detection. HIV-1 infection was identified by positive anti-HIV responses. All recruited patients were negative for hepatitis B surface antigen (HBsAg) and had never received any forms of HCV-specific antiviral therapy.

All HCV/HIV-1-coinfected patients had previously received regular or intermittent HIV-specific treatment using highly active antiretroviral therapy (HAART) consisting of two nucleoside reverse transcriptase inhibitors (NRTIs): azidothymidine (AZT) plus didanosine (ddI) (~60%); or stavudine (d4T) plus lamivudine (3TC) (~40%), and one non-nucleoside reverse transcriptase inhibitor (NNRTI): nevirapine (NVP). The treatment was provided through support from the China CARES (Community AIDS Resource and Education Services) program. All participants were interviewed by trained and qualified staff using a standardized questionnaire, including detailed general information, blood donation history, and usage of antiviral or antiretroviral drugs. The clinical backgrounds of the 139 followed-up patients are shown in Additional file 1: Table S1. The study was approved by the institutional review authorities of Peking University Health Science Center (Approval ID: PKUPHLL20090011). All patients provided written informed consent before enrollment in the study.

### Sample collection and clinical evaluation

Serum and EDTA anticoagulant plasma were immediately stored in -80°C after collection. Several liver function indexes, alanine aminotransferase (ALT), aspartate aminotransferase (AST), and γ glutamyl transpeptidase (γ-GT), total protein, albumin and bilirubin, were measured by traditional clinical standardized methods on Unicer Dxc 800 Synchron Clinical System (Beckman Coulter, Fullerton, CA, USA). Also, the fatty liver grades of recruited patients were evaluated by ultrasonography [20].

### HCV/HIV seropositive screening and confirmation

Plasma HCV antibody was detected using the Abbott Architect anti-HCV assay (Abbott GmbH & Co KG, Wiesbaden, Germany); Anti-HCV status of all positive anti-HCV were confirmed by HCV-RIBA assay (Wantai Biological Pharmacy), which utilizes recombinant proteins (Core, NS3, NS4 and NS5) immobilized as individual bands onto test strips. The intensity of the HCV bands is scored in relation to the intensities of the internal IgG controls as 5 different levels: 0(none), +, ++, +++, ++++, according to the manufacturer's instructions. The HIV-1 screening test was performed in the local center of diseases control and prevention (CDC) and was based on HIV-1 antibodies ELISA assay (GBI Biotech Co., Ltd., Beijing, China) and all positive tests were confirmed by HIV Blot 2.2 WB assay (HIV Blot 2.2 WB; Genelabs Diagnostics, Singapore).

### HCV core antigen quantification

Plasma HCV-coreAg quantification was performed using a commercial chemiluminescent microparticle immunoassay (CMIA) (ARCHITECT HCV Ag Reagent Kit, Abbott Diagnostics, Abbott Park, USA) with a detection limit of 3 fmol/L, according to the manufacturer's instructions.

### HCV-RNA quantification

Plasma HCV-RNA measurements were performed using the Abbott Real-Time HCV Amplification Kit (Abbott Molecular Inc. Des Plaines, IL, USA) according to the manufacturer's instructions. The detection limit was 30 IU/mL, equivalent to 1.48 log_10_ IU/mL, with the 0.2 mL sample preparation procedure.

### CD4+/CD8+ T-cell counts

All reagents were purchased from BD Biosciences (BD Biosciences, San Jose, CA) and CD4+/CD8+ T-cell counts were measured within 12 hours using a FACSCalibur (BD Biosciences, San Jose, CA).

### *In vitro*HCV particle degradation experiment

Plasma samples were collected from seven HIV-1-monoinfected patients and eight healthy individuals, with their informed consent, in 2012. For each patient, 3 mL plasma was equally divided into three 1.5 mL microtubes. Then, 200 μL HCVcc stock (HCV-JFH1 virus, 1 × 10^7^ copies/mL) was harvested from the cell culture supernatant of JFH1-tansfected Huh 7.5.1 cells (maintained in Dulbecco's modified Eagle's medium [DMEM] supplemented with 10% fetal bovine serum), and was added into each 1.5 mL microtube. DMEM medium was used as the blank control. HIV-1-infected plasma samples (and medium-only controls) were incubated in a CO_2_ incubator at 37°C for 0 and 12 hours, separately, and then frozen immediately until testing for HCV-coreAg and HCV-RNA. The clinical backgrounds of the seven HIV-1-monoinfected patients and eight healthy individuals are shown in Additional file 2: Table S2.

### Statistical analysis

Descriptive statistics shown were the median with 25% and 75% percentiles, as appropriate. Comparisons between groups were conducted using unpaired *t* tests or Mann Whitney U-tests. All statistical analyses were performed using GraphPad Prism for Windows, version 5.0 (GraphPad Software Inc., San Diego, CA). All p-values were two-tailed, and were considered significant when lower than 0.05.

## Results

### The concentration of HCV-coreAg was highly correlated with HCV-RNA levels in HCV-monoinfected and HCV/HIV-1-coinfected patients over 3-year observation

**Figure 2:**
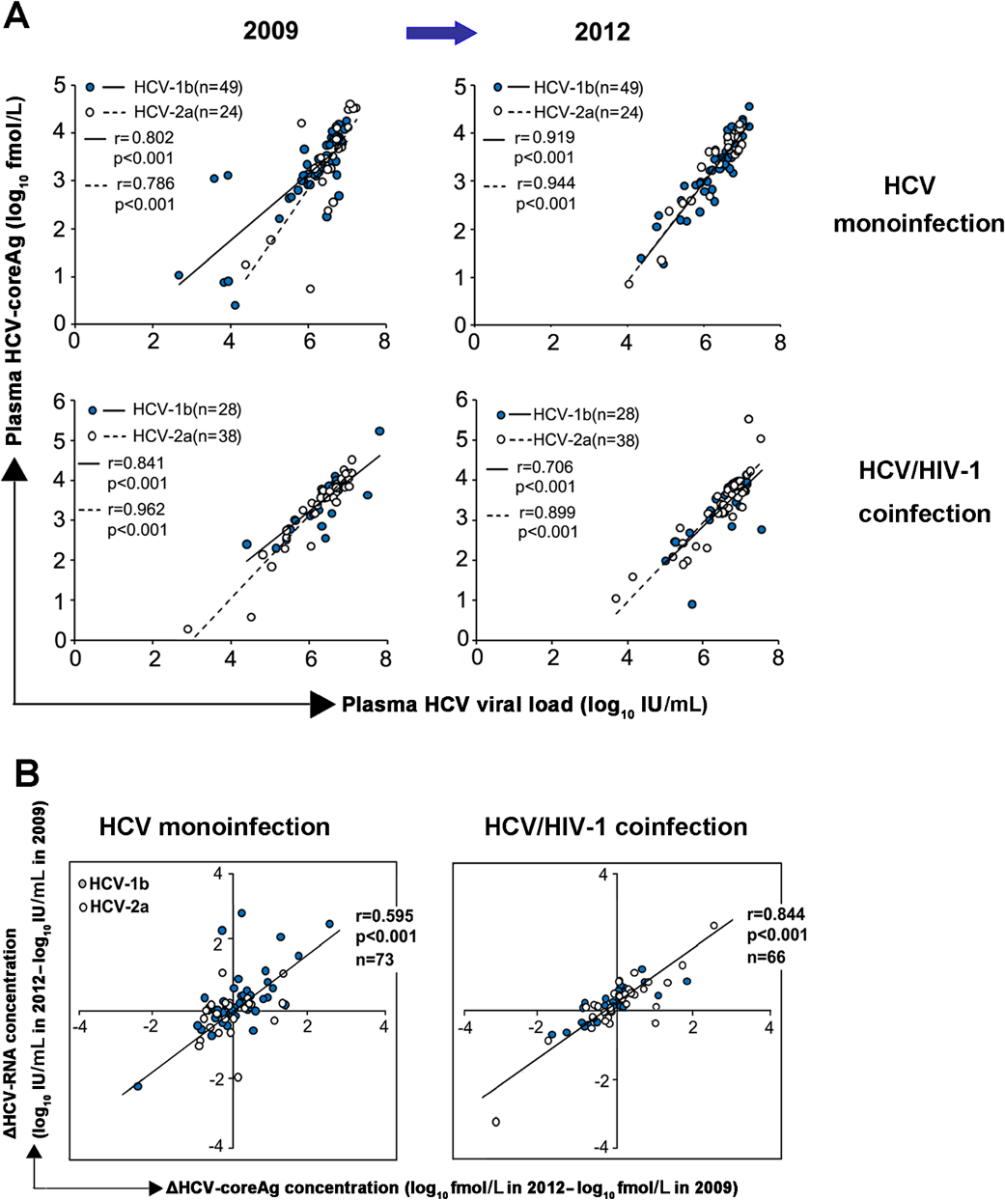
**The correlation was showed between the concentration of hepatitis C virus core antigen (HCV-coreAg) and HCV-RNA at two time points. (A)** The levels of HCV-coreAg were highly correlated with serum HCV-RNA load, both in HCV-monoinfected and HCV/HIV-1-coinfected patients in 2009 and 2012, for both HCV genotypes 1b (●) and 2a (○). **(B)** Correlation of ΔHCV-coreAg concentration (log_10_fmol/L in 2012 - log_10_fmol/L in 2009) and ΔHCV-RNA concentration (log_10_IU/mL in 2012 - log_10_IU/mL in 2009) at two time points both in HCV-monoinfected and HCV/HIV-1-coinfected patients. Spearman's rank-correlation test was performed. All values are log_10_-transformed, p < 0.05 indicates significance.

The present study confirmed that, in HCV-monoinfected patients, HCV-coreAg and HCV-RNA were significantly correlated at the two different time points (2009, HCV-1b: r = 0.802, p < 0.001, HCV-2a: r = 0.786, p < 0.001; 2012, HCV-1b: r = 0.919, p < 0.001, HCV-2a: r = 0.944, p < 0.001, Figure 2A). Similarly, HCV-RNA and HCV-coreAg were also correlated, at both time points, in HIV-1-coinfected patients (2009, HCV-1b: r = 0.841, p < 0.001; HCV-2a: r = 0.962, p < 0.001; 2012, HCV-1b: r = 0.706, p < 0.001; HCV-2a: r = 0.899, p < 0.001, Figure 2A). In addition, 100% of the HCV-RNA positive samples were also positive by the HCV-coreAg assay (data not shown).

### The dynamics of the changes in HCV-RNA and HCV-coreAg at two time points

To clearly reflect the dynamics of the correlation between HCV-RNA and HCV-coreAg at two different time points, the differences in HCV-coreAg concentration (ΔHCV-coreAg = concentration at 2009-concentration at 2012) and HCV-RNA levels (ΔHCV-RNA = concentration at 2009-concentration at 2012) were calculated and analyzed. As shown in Figure 2B, a strong correlation (r = 0.595, p < 0.001) was seen between ΔHCV-RNA and ΔHCV-coreAg in patients with HCV monoinfection, and a similar positive correlation was observed in patients with HCV/HIV-1 coinfection (r = 0.844, p < 0.001). This result suggested that HCV-coreAg and HCV-RNA changed in parallel with each other over the 3-year observation.

### Negative correlation between the ratios of HCV-coreAg to HCV-RNA and CD4+ T-cell counts in HCV/HIV-1-coinfected patients

**Figure 3:**
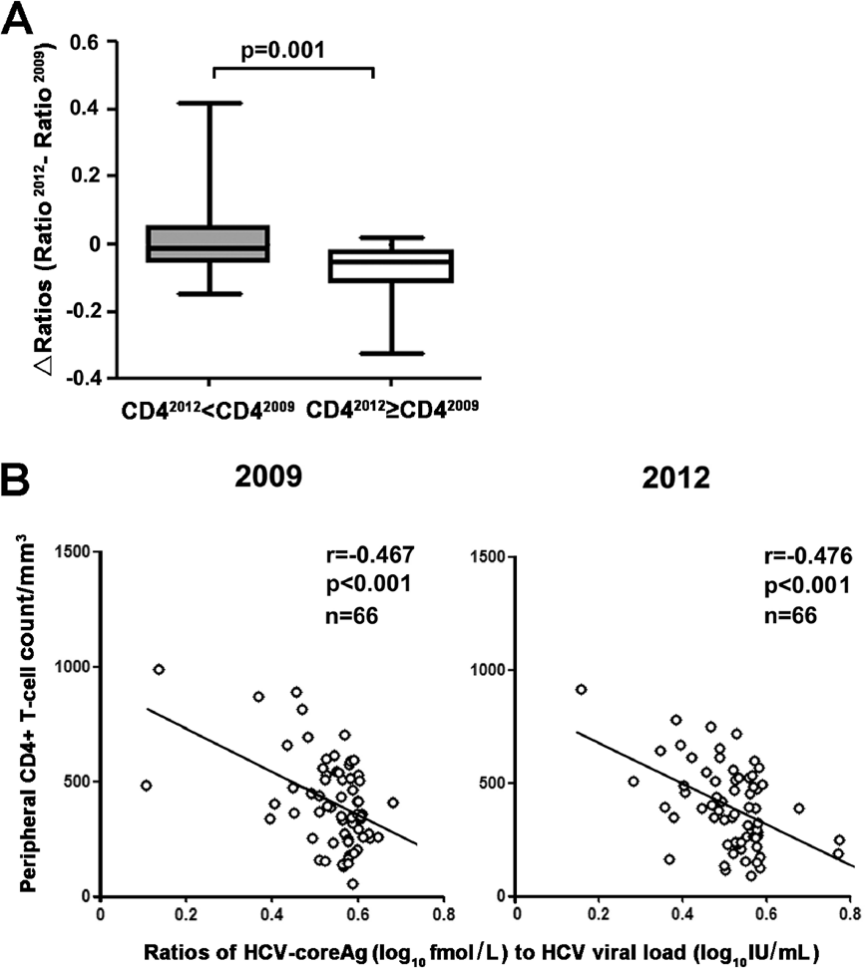
**HIV-related immune dysfunction associated with the ratio of HCV-coreAg to HCV-RNA (Ag/RNA).** Negative correlation between the ratio of HCV-coreAg to HCV-RNA (Ag/RNA) and CD4+ T-cell counts in HCV/HIV-1-coinfected patients. **(A)** ΔAg/RNA ratios (ratio^2012^ - ratio^2009^) were significantly lower in HCV/HIV-1-coinfected patients with CD4^2012^≥ CD4^2009^ than in those with CD4^2012^ < CD4^2009^. **(B)** Negative correlations between CD4+ T-cell counts and Ag/RNA ratios were seen both in 2009 and 2012. Mann Whitney U-tests and Spearman's rank-correlation were performed in **(A)** and **(B)**, separately, p < 0.05 indicates significance.

Next, we explored the association between the ratio of the HCV-coreAg to the HCV-RNA (Ag/RNA) and immune status in HCV/HIV-1-coinfected patients. Interestingly, when the HCV/HIV-1-coinfected patients were divided into two groups according to variation in CD4+ T-cell counts at the two time points, the patients with CD4^2012^≥ CD4^2009^ showed lower ΔAg/RNA ratios (ratio^2012^-ratio^2009^) than patients with CD4^2012^ < CD4^2009^ (p = 0.001, Figure 3A). Furthermore, significant negative correlations were observed between Ag/RNA ratios and CD4+ T-cell counts both in 2009 and 2012 (2009: r = -0.467, p < 0.001; 2012: r = 0.476, p < 0.001, Figure 3B).

### HCV particle degradation *in vitro*

The data presented above showed that the Ag/RNA ratios were negatively correlated with CD4+ T-cell counts. It is conceivable that the higher degradation rates of HCV-RNA and/or the slower decay rate of HCV-coreAg under the HIV-related immunocompromised condition may be partially responsible for this. To support our hypothesis, an *in vitro* experiment investigating the possibility of degradation of HCV particles was conducted. The experiment was performed within the environment of healthy and HIV-1-positive plasma as shown in "materials and methods". These conditions mimic HCV-monoinfected and HCV/HIV-1-coinfected plasma, respectively, and are therefore suited to evaluate the degradation dynamics of the HCV core protein and RNA during incubation at 37°C for 12 hours.

**Figure 4:**
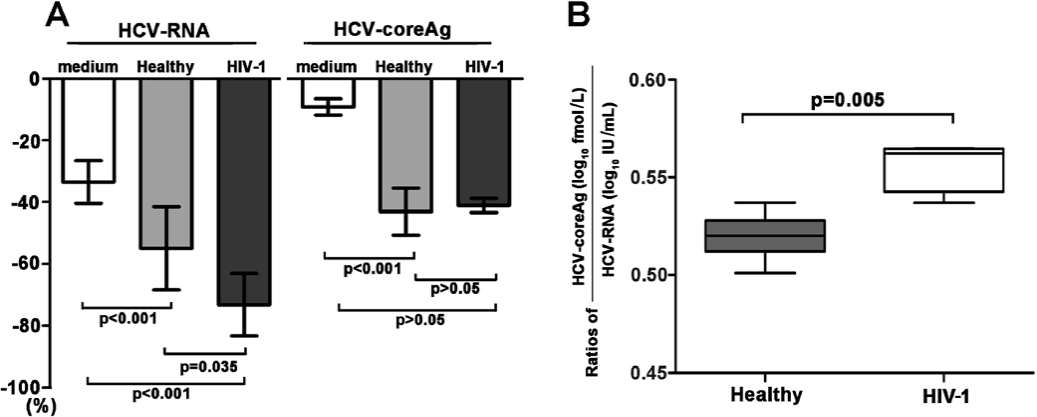
***In vitro*** **hepatitis C virus particle degradation experiment** ***.*** Changes in the degradation dynamics of HCV-RNA and HCV- coreAg, **(A)** in medium, HIV-1-positive, and healthy plasma after incubation at 37°C for 12 hours. The X axis indicates the degradation of HCV-RNA or HCV-coreAg at 12 hours, expressed as a percentage of the initial levels. **(B)** HCV-coreAg/HCV-RNA ratios at 12 hours in HIV-1-positive plasma were significantly higher than those in healthy plasma. Unpaired t tests and Mann Whitney U-tests were performed, p < 0.05 indicates significance.

After incubating at 37°C for 12 hours, the average HCV-RNA levels decreased to 33.49%, 54.92%, and 73.16% of the initial levels in medium, healthy plasma, and HIV-1-positive plasma, respectively, whereas at the same time, the average HCV-coreAg levels decreased to 9.16%, 46.16%, and 42.45% of the initial levels. Decay rates of HCV-RNA and HCV-coreAg at 12 hours were significantly lower in medium than in healthy plasma (HCV-RNA: p < 0.001, HCV-coreAg: p < 0.001) and in HIV-1-positive plasma (HCV-RNA: p < 0.001, HCV-coreAg: p < 0.001) (Figure 4A). The decay rate of HCV-RNA in healthy plasma was significantly lower than in HIV-1-positive plasma (p = 0.035), while no similar difference was observed in the decay of HCV-coreAg (p > 0.05). Further, we observed that the Ag/RNA ratios in HIV-1-positive plasma were significantly higher than in healthy plasma (p = 0.005, Figure 4B). Certainly, it must be noted that the influence of blood cells on degradation of HCV is not addressed in this *in vitro* experiment.

### Detection and analysis of anti-HCV antibody response

**Figure 5:**
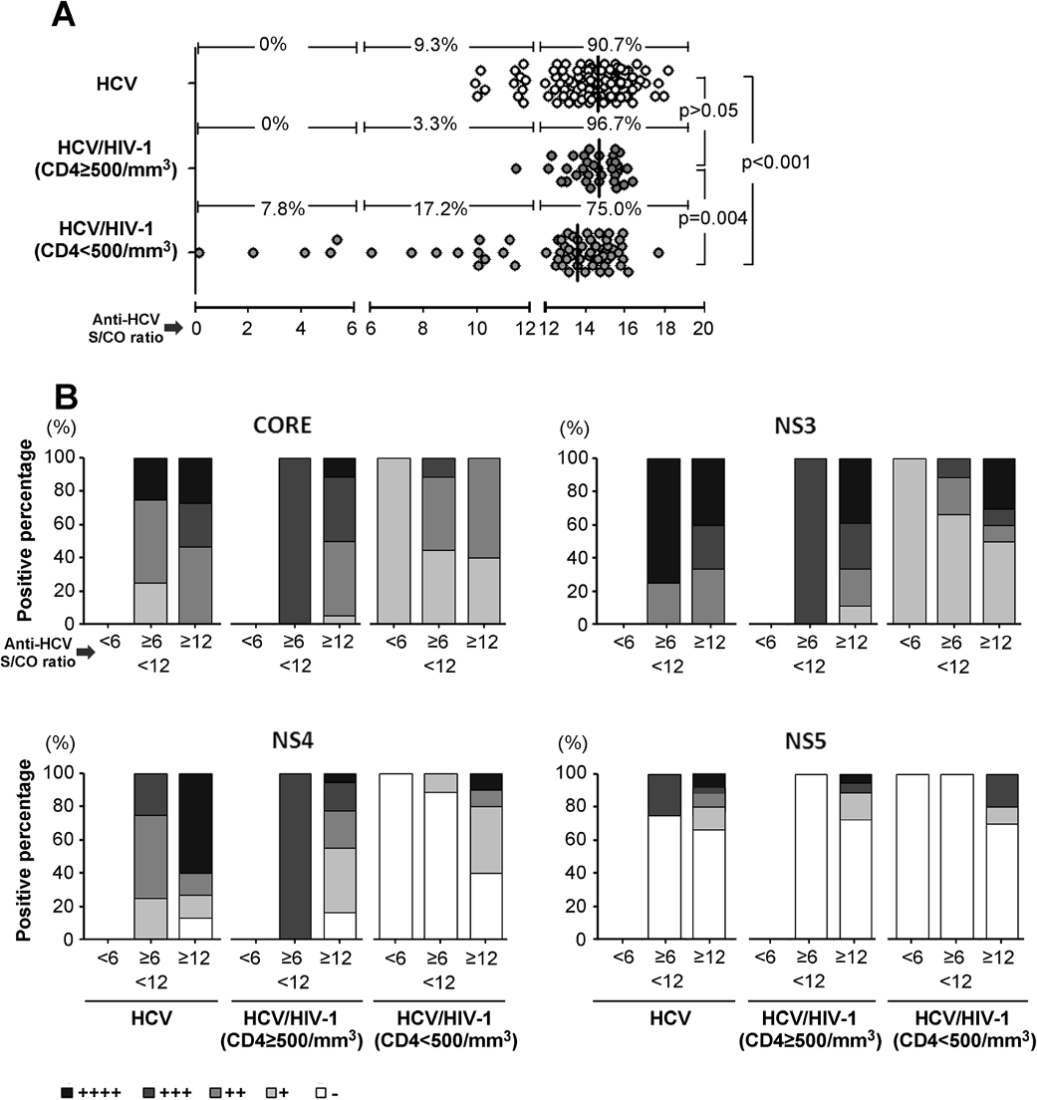
**Anti-HCV status of HCV monoinfection, HCV/HIV coinfection with CD4 ≥ 500/mm** ^**3**^ **and coinfection with CD4 < 500/mm** ^**3**^ **were performed by Architect anti-HCV assay and HCV-RIBA assay respectively. (A)** S/CO ratios of anti-HCV antibody of HCV/HIV-1-coinfected patients with CD4+ T <500/mm^3^ were significantly lower than coinfected patients with CD4+ T ≥500/ mm^3^and HCV-monoinfected patients. **(B)** Anti-HCV responses induced by Core, NS3, NS4 and NS5 proteins of HCV monoinfection, HCV/HIV coinfection with CD4 ≥ 500/mm^3^ and coinfection with CD4 < 500/mm^3^ were retested by HCV-RIBA assay. The intensity of the HCV bands is scored in relation to the intensities of the internal IgG controls as 5 different levels: 0(none), +, ++, +++, ++++, according to the manufacturer's instructions. Mann Whitney U-tests were performed, p < 0.05 indicates significance.

In the Figure 5, the comparison of the anti-HCV Ab among three groups of recruited participants in 2009 survey (including 129 HCV-monoinfected subjects, 34 HCV/HIV-1 coinfected subjects with CD4 ≥ 500/mm^3^ and 64 coinfected subjects with CD4 < 500/mm^3^) were performed by Architect anti-HCV assay and HCV-RIBA assay respectively. As shown in Figure 5A, the anti-HCV Ab titer of coinfection with CD4 < 500/mm^3^ was the lowest level among three groups (p < 0.001 and p = 0.004, separately). Also, the HCV-RIBA data (Figure 5B) indicated that anti-HCV responses induced by Core, NS3, NS4 and NS5 proteins were highest in HCV monoinfected patients, to a less degree found in HCV/HIV coinfection with CD4 ≥ 500/mm^3^ and lowest in coinfection with CD4 < 500/mm^3^.

## Discussion

Several studies conclude that HCV-coreAg determination using the ARCHITECT platform would be a useful strategy for monitoring HCV infection and discriminating periods with active viral replication from spontaneous clearances. This approach could be used as a supplement to response-guided antiviral therapy [13],[15]. In our study, all HCV-RNA-positive samples were also positive for HCV-coreAg, giving a detection rate of 100% by HCV-coreAg. In accordance with the published data [9],[13],[15],[21], our data also showed a comparatively high diagnostic sensitivity for the HCV-coreAg quantitative assay.

In our longitudinal study, high correlations between HCV-RNA and HCV-coreAg were observed in both HCV-monoinfected and HCV/HIV-1-coinfected patients, for both genotypes 1b and 2a of HCV. These observations confirm results from other published data based on the different cross-sectional studies [9],[15]-[17],[21]-[23]. Our findings also suggested that the correlation between HCV-coreAg and HCV-RNA is relatively stable in the same group, even at different time points. Taken together, the HCV-coreAg assay is a feasible alternative method for HCV-RNA determination.

It is believed that the amount of HCV-RNA is constantly proportional to the amount of HCV-coreAg in complete viral particles. Christian et Al. [24] suggested that variability in the ratio of HCV-RNA and core protein could be an additional indicator for treatment follow-up, as well as providing information on HCV replication. Magali et al. [12] found that different HCV-monoinfected individuals had minor differences in the amount of core protein and HCV-RNA. Mederache et al. found that no significant difference in HCV-RNA/HCV-coreAg ratios between HIV-1/HCV-, HBV/HCV-, and HCV-infected hemodialysis patients [25]. However, the characteristics of variable Ag/RNA ratios in different HCV/HIV-1-coinfected patients are not well understood. In present 3-year longitudinal study, we observed higher Ag/RNA ratios in HCV/HIV-1-coinfected patients with lower CD4+ T-cell counts. Moreover, the Ag/RNA ratios were found to be negatively correlated with CD4+ T-cell counts in HCV/HIV-1-coinfected patients. These results indicated that differences in the levels of core protein and HCV-RNA are broadened in HCV/HIV-1-coinfected patients with declining CD4+ T-cell counts, though the underlying mechanism remains unclear. Some possible explanations are related to the idea that the kinetics of degradation of HCV-coreAg and HCV-RNA in HCV/HIV coinfection differ from those of HCV monoinfection. Accumulated literatures [26]-[29] have demonstrated that microbial translocation is a cause of systemic immune activation in chronic HIV infection, which characterized by higher levels of plasma bacterial DNA and LPS in HIV-infected patients compared to healthy controls. Considering such phenomenon, we hypothesized that the disintegrated bacteria would probably release much more RNase into plasma of immunosuppressed HIV subjects. Consistent with this theory, our *in vitro* data supported the idea that the greater instability of HCV-RNA in HIV plasma may partly account for the higher Ag/RNA ratios in HCV/HIV-1-coinfected patients with lower CD4+ T-cell counts. Additionally, as shown in Figure 5, HCV/HIV-1-coinfected patients with an impaired immune system may produce fewer antibodies against HCV-coreAg than HCV-monoinfected patients. It is conceivable that anti-core antibodies might interfere with the detection of HCV-coreAg or shorten its half-life in circulation, which could be an alternative explanation for the different Ag/RNA ratios seen in coinfected patients. Thus, based on present observations, serum HCV-Ag quantitation might more reliably reflect the authentic level of HCV copies than HCV-RNA determination in HCV/HIV-coinfected patients with compromised immunity. However, we could neither prove nor disprove this hypothesis through the experiment described here, because the HIV-positive and healthy plasma did not contain anti-HCV antibodies. On the other hand, other plasma components, such as viral proteins, lipids, and microRNA may also affect the decay of HCV-RNA and core protein.

## Conclusions

In conclusion, our longitudinal study indicated that the HCV-coreAg presented comparable dynamics as HCV RNA over time in chronic HCV-infected patients. These data also suggested that the Ag/RNA ratio had unique characteristics that varied among different infectious statuses and was negatively correlated with CD4+ T-cell count in HCV/HIV-1-coinfected patients.

## Authors' contributions

TS and FL conceived the study, drafted the experimental protocol and manuscript. LL, JG and ZD performed the experiments and analyzed the data. TS and LL and HL contributed to writing, reviewing, and revising the paper. All authors interpreted the data and critically reviewed drafts of the manuscript. All authors edited and approved the final manuscript.

## Additional files

## Electronic supplementary material

Additional file 1: Table S1.: The clinical characteristics of 73 HCV-monoinfected and 66 HCV/HIV-1-coinfected patients enrolled in our longitudinal study. (DOC 103 KB)

Additional file 2: Table S2.: The clinical characteristics of 7 HIV-1-monoinfected patients and 8 healthy individuals enrolled in the *in vitro* study. (DOC 58 KB)

Below are the links to the authors’ original submitted files for images.

Authors’ original file for figure 1

Authors’ original file for figure 2

Authors’ original file for figure 3

Authors’ original file for figure 4

Authors’ original file for figure 5

## Abbreviations

HCV: Hepatitis c virus
HIV: Human immunodeficiency virus
HCV-coreAg: Hepatitis c virus core antigen
HBsAg: Hepatitis B surface antigen
Ab: antibody
HAART: Highly active antiretroviral therapy
NRTIs: Nucleoside reverse transcriptase inhibitors
AZT: Azidothymidine
ddI: Didanosine
d4T: Stavudine
NNRTI: Non-nucleoside reverse transcriptase inhibitor
NVP: Nevirapine
ALT: Alanine aminotransferase
AST: Aspartate aminotransferase
γ-GT: γ glutamyl transpeptidase
CMIA: Chemiluminescent microparticle immunoassay
