# Supplementary material for: Effectiveness of HCV core antigen and RNA quantification in HCV-infected and HCV/HIV-1-coinfected patients
Source: BMC Infect Dis. 2014 Nov 5;14:577. doi: 10.1186/s12879-014-0577-1 (PMC4225041; doi:10.1186/s12879-014-0577-1)
Supplement: Supplementary file 1 — Additional file 1: Table S1.: The clinical characteristics of 73 HCV-monoinfected and 66 HCV/HIV-1-coinfected patients enrolled in our longitudinal study. (DOC 103 KB) [file 12879_2014_577_MOESM1_ESM.doc]

**Additional file 1: Table S1. The clinical characteristics of 73 HCV-monoinfected and 66 HCV/HIV-1-coinfected patients enrolled in our longitudinal study.**

|  | **HCV-monoinfected (n=73)** | | **HCV/HIV-1-coinfected (n=66)** | |
| --- | --- | --- | --- | --- |
| **Characteristic** | 2009 | 2012 | 2009 | 2012 |
| Age (years), median (IQR) | 52.6(42.5-59.8) |  | 47.9(39.6-54.5) |  |
| Gender, n(%) |  | | | |
| Male | 32 (43.8) |  | 32 (48.5) |  |
| Female | 41 (56.2) |  | 34 (51.5) |  |
| HBsAg | negative | negative | negative | negative |
| anti-HIV | negative | negative | positive | positive |
| anti-HCV S/CO value, median (IQR) | 14.4(13.6-15.4) | 14.2(12.8-15.7) | 14.4(12.9-15.5) | 14.3(13.1-15.8) |
| HCV RNA(log_10_ IU/mL), median (IQR) | 6.3(5.8-6.6) | 6.4(5.8-6.5) | 6.5(5.9-6.8) | 6.1(5.7-6.7) |
| HCV core Ag(log_10_ fmol/L), median (IQR) | 3.2(2.5-4.7) | 3.3(2.4-4.7) | 3.3(2.6-4.6) | 3.2(2.5-4.6) |
| HCV genotype, n(%) |  |  |  |  |
| 1b | 50 (68.5) |  | 28 (42.4) |  |
| 2a | 23(31.5) |  | 38 (57.6) |  |
| Others | none |  | none |  |
| CD4+ T-cell counts, median (IQR) | N.A. | N.A. | 415(294-558) | 418(287-560) |
| CD8+ T-cell counts, median (IQR) | N.A. | N.A. | 963(680-1157) | 951(662-1240) |
| Blood routine examination |  |  |  |  |
| WBC (10^9^/L), median (IQR) | 6.2(4.9-7.7) | 6.3(5.2-7.6) | 5.6(4.4-7.1) | 5.2(4.0-6.8) |
| RBC (10^12^/L), median (IQR) | 5.2(4.8-5.7) | 5.2(4.4-5.8) | 4.3(3.7-4.9) | 4.0(3.5-4.7) |
| Hemoglobin (g/L), median (IQR) | 143.1(128.2-154.5) | 138.2(125.4-155.2) | 127.0(113.5-146.0) | 119.5(114.3-142.1) |
| Platelet (10^9^/L), median (IQR) | 222.0(167.3-251.8) | 210.6(155.7-259.2) | 151.5(115.3-194.8) | 142.0(107.5-187.5) |
| Biochemistry analysis |  |  |  |  |
| ALT (IU/L), median (IQR) | 33.2(25.1-54.2) | 33.3(26.1-55.6) | 37.6(24.9-60.5) | 37.8(23.9-58.4) |
| AST(IU/L), median (IQR) | 37.7(26.1-61.1) | 39.8(25.9-64.5) | 38.2(21.6-65.4) | 38.5(22.1-64.2) |
| Total protein (g/L), median (IQR) | 77.8(73.4-79.2) | 76.4(72.8-78.6) | 77.5(74.2-83.6) | 77.1(74.1-82.6) |
| Albumin (g/L), median (IQR) | 44.2(39.6-48.4) | 44.1(40.2-49.5) | 44.6(40.9-48.7) | 43.2(39.4-47.9) |
| Total bilirubin (μmol/L), median (IQR) | 14.1(11.2-16.9) | 14.3(10.8-17.2) | 13.0(10.9-16.8) | 12.8(10.2-15.4) |
| Direct bilirubin (μmol/L), median (IQR) | 4.3(3.3-5.4) | 4.4(3.2-5.5) | 4.2(3.1-5.6) | 4.4(3.1-5.7) |
| BMI, median (IQR) | 23.1(21.0-25.4) | 23.0(20.8-25.6)) | 22.5(20.8-24.8) | 22.7(20.4-25.0) |
| APRI, median (IQR) | 0.19(0.10-0.37) | 0.18(0.11-0.36) | 0.22(0.14-0.58) | 0.23(0.13-0.59) |
| Hepatitis, n(%) | 15(20.5) | 17(23.3) | 20(30.3) | 22(33.3) |
| Fatty liver, n(%) |  |  |  |  |
| none | 69(94.5) | 67(91.8) | 59(89.4) | 59(89.4) |
| Low-grade | 4(5.5) | 6(8.2) | 7(10.6) | 7(10.6) |
| Medium-grade | 0(0) | 0(0) | 0(0) | 0(0) |
| Cirrhosis, n(%) | 1(1.37) | 1(1.37) | 1(1.52) | 2(3.03) |
| Liver cancer, n(%) | 0(0) | 0(0) | 0(0) | 0(0) |
| Cholecystitis, n(%) | 18(24.66) | 18(24.66) | 20(30.30) | 20(30.30) |
| Hypertension, n(%) | 9(12.33) | 13(17.8) | 10(15.15) | 14(21.2) |
| Diabetes, n(%) | 2(2.74) | 2(2.74) | 3(4.54) | 3(4.54) |
| Heperlipidemia, n(%) | 8(10.96) | 10(13.7) | 7(10.61) | 8(12.1) |
| Hepatic nephropathy, n(%) | 1(1.37) | 2(2.74) | 2(3.03) | 3(4.56) |
| Blood donation History |  |  |  |  |
| FBDs, n(%) | 69 (94.52) |  | 65 (98.48) |  |
| Non-FBDs, n(%) | 4 (5.48) |  | 1 (1.52) |  |
| HCV-specific therapy | none | none | none | none |
| HAART |  |  |  |  |
| Occasional, n(%) | N.A. | N.A. | 2(3.03) | 2(3.03) |
| Intermittent, n(%) | N.A. | N.A. | 4(6.06) | 4(6.06) |
| Regular, n(%) | N.A. | N.A. | 58(87.34) | 58(87.34) |
| Unclear, n(%) | N.A. | N.A. | 2(3.03) | 2(3.03) |

IQR: interquartile range; ALT: Alanine Aminotransferase; AST: aspartate aminotransferase; FBD: former blood donors; HAART: high active antiretroviral therapy; APRI: Aspartate aminotransferase to platelet ratio index; BMI: body mass index, calculated as the weight in kilograms divided by the square of height in meters; N.A.: not available.
