# Supplementary material for: Effectiveness of HCV core antigen and RNA quantification in HCV-infected and HCV/HIV-1-coinfected patients
Source: BMC Infect Dis. 2014 Nov 5;14:577. doi: 10.1186/s12879-014-0577-1 (PMC4225041; doi:10.1186/s12879-014-0577-1)
Supplement: Supplementary file 2 — Additional file 2: Table S2.: The clinical characteristics of 7 HIV-1-monoinfected patients and 8 healthy individuals enrolled in the in vitro study. (DOC 58 KB) [file 12879_2014_577_MOESM2_ESM.doc]

**Additional file 2: Table S2. The clinical characteristics of 7 HIV-1-monoinfected patients and 8 healthy individuals enrolled in the *in vitro* study.**

|  | **HIV-1-monoinfected (n=7)** | **Healthy (n=8)** |
| --- | --- | --- |
| **Characteristic** | 2009 | 2012 |
| Age (years), median (IQR) | 38(31-43) | 40.5(31-41.8) |
| Gender, n(%) |  |  |
| Male | 4 (57.2) | 4(50) |
| Female | 3 (42.8) | 4(50) |
| HBsAg | negative | negative |
| anti-HIV | positive | negative |
| anti-HCV | negative | negative |
| HCV RNA | negative | negative |
| CD4+ T-cell counts, median (IQR) | 307(218.5-518.5) | N.A. |
| CD8+ T-cell counts, median (IQR) | N.A. | N.A. |
| Blood routine examination |  |  |
| WBC (10^9^/L), median (IQR) | 6.9(4.65-7.7) | N.A. |
| RBC (10^12^/L), median (IQR) | 4.45(3.14-4.5) | N.A. |
| Hemoglobin (g/L), median (IQR) | 108(79-156.5) | N.A. |
| Platelet (10^9^/L), median (IQR) | 265(206.5-386.5) | N.A. |
| Biochemistry analysis |  |  |
| ALT (IU/L), median (IQR) | 17(13.5-20) | N.A. |
| AST(IU/L), median (IQR) | 23(21.5-34.5) | N.A. |
| Total protein (g/L), median (IQR) | 80.6(77.4-89.9) | N.A. |
| Albumin (g/L), median (IQR) | 47.2(44-53.8) | N.A. |
| Total bilirubin (μmol/L), median (IQR) | 13.6(12.5-19.7) | N.A. |
| Direct bilirubin (μmol/L), median (IQR) | 5.7(4.1-7.0) | N.A. |
| BMI, median (IQR) | 23.7(21.6-26.5) | N.A. |
| APRI, median (IQR) | 0.09(0.06-0.16) | N.A. |
| Blood donation History |  |  |
| FBDs, n(%) | 3(42.8) | N.A. |
| Non-FBDs, n(%) | 4(57.2) | N.A. |
| HAART |  |  |
| Occasional, n(%) | N.A. | N.A. |
| Intermittent, n(%) | 1(14.3) | N.A. |
| Regular, n(%) | 4(57.1) () | N.A. |
| Unclear, n(%) | 2(28.6) | N.A. |

IQR: interquartile range; ALT: Alanine Aminotransferase; AST: aspartate aminotransferase; FBD: former blood donors; HAART: high active antiretroviral therapy; BMI: body mass index, calculated as the weight in kilograms divided by the square of height in meters; N.A.: not available.
