# Supplementary figures and images for: Effectiveness of HCV core antigen and RNA quantification in HCV-infected and HCV/HIV-1-coinfected patients
Source: BMC Infect Dis. 2014 Nov 5;14:577. doi: 10.1186/s12879-014-0577-1 (PMC4225041; doi:10.1186/s12879-014-0577-1)

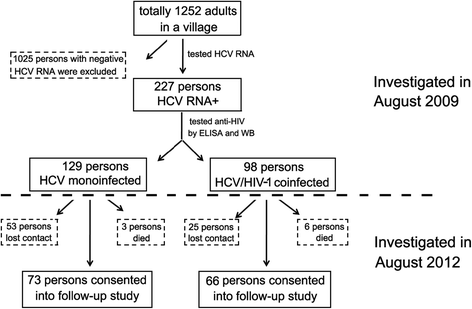

Supplement: Supplementary file 3 — Authors’ original file for figure 1 [file 12879_2014_577_MOESM3_ESM.gif]

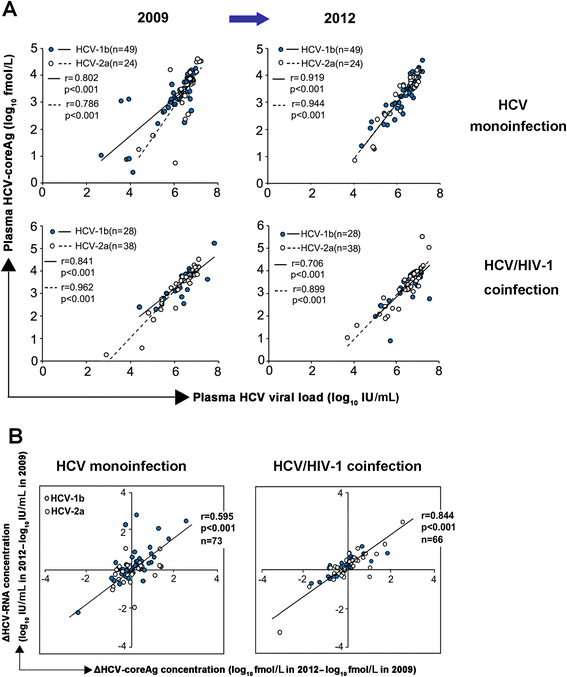

Supplement: Supplementary file 4 — Authors’ original file for figure 2 [file 12879_2014_577_MOESM4_ESM.gif]

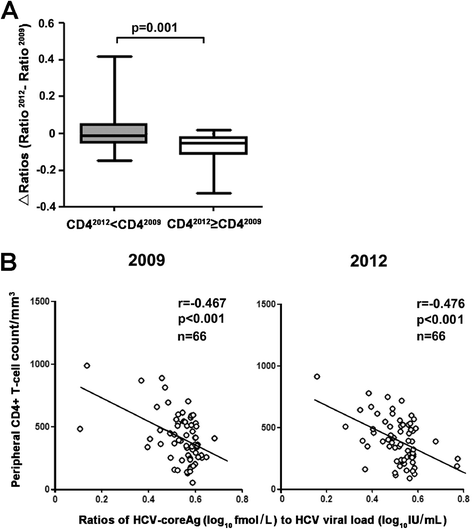

Supplement: Supplementary file 5 — Authors’ original file for figure 3 [file 12879_2014_577_MOESM5_ESM.gif]

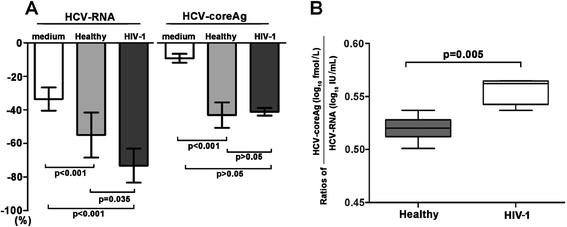

Supplement: Supplementary file 6 — Authors’ original file for figure 4 [file 12879_2014_577_MOESM6_ESM.gif]

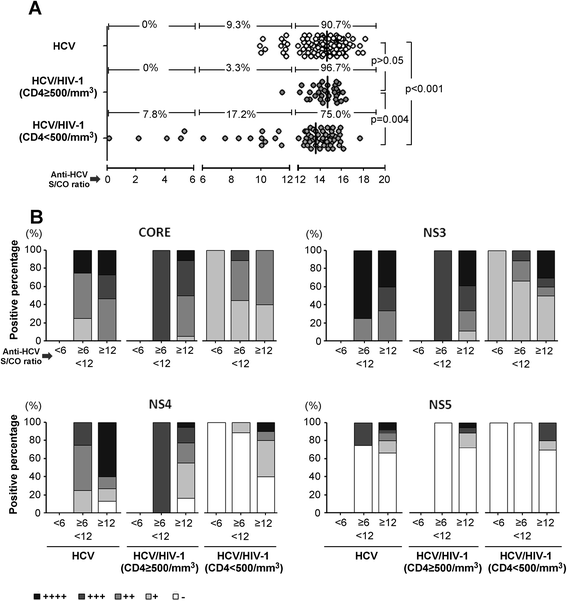

Supplement: Supplementary file 7 — Authors’ original file for figure 5 [file 12879_2014_577_MOESM7_ESM.gif]
